# Supplementary material for: Exploring the Cause of Diarrhoea and Poor Growth in 8–11-Week-Old Pigs from an Australian Pig Herd Using Metagenomic Sequencing
Source: Viruses. 2021 Aug 13;13(8):1608. doi: 10.3390/v13081608 (PMC8402840; doi:10.3390/v13081608)
Supplement: Supplementary file 1 [file viruses-13-01608-s001.zip › Supplementary Materials Porcine Viruses Manuscript 22 July 2021.pdf]

Supplementary Information

Exploring the cause of diarrhoea and poor growth in 8-11 weeks old pigs from an Australian pig herd using metagenomics sequencing

Tarka Raj Bhatta [1,2], Anthony Chamings [1,2], Soren Alexandersen*[1,2,3]

[1]Geelong Centre for Emerging Infectious Diseases, Geelong, VIC 3220, Australia; [2]Deakin University, School of Medicine, Geelong, VIC 3220, Australia; [3]Barwon Health, Geelong, VIC 3220 Australia

*Corresponding Author: soren.alexandersen@deakin.edu.au

Supplementary Table S1. Table showing the abundance of bacterial chromosomal DNA/plasmid reads in different pig swab samples.

	**Percentage abundance of reads among total bacterial reads and also among total NGS reads (shown inside bracket)**			
	**Samples**			
	**Pig 45, Colon**	**Pig 45, Lung**	**Pig 46, Colon**	**Pig 46, Lung**
	**PC45-BC24**	**PL45-BC23**	**PC46-BC26**	**PL46-BC25**
**Bacteria name**				
Lawsonia intracellularis	2.05 (0.0062)	2.08 (0.0097)	7.2 (0.0469)	0.25 (0.0032)
Brachyspira intermedia	0.12 (0.0004)	0.12 (0.0006)	0.59 (0.0039)	-
Brachyspira hyodysenteriae	0.05 (0.0002)	0.05 (0.0002)	0.11 (0.0007)	-
Campylobacter spp	0.5 (0.002)	0.5 (0.0024)	0.16 (0.0011)	0.06 (0.0008)
**All mapped bacterial reads of total reads (percentage)**	**18110 (0.3045)**	**39053 (0.4682)**	**45420 (0.6530)**	**36605 (1.2908)**

Supplementary Table S2. Ten consensus partial sequences of porcine sapelovirus (PSV) were obtained from Pig45 and Pig46 colon and lung swab samples. The values for each sequence length, IGV coverage, mapping quality and accession number are shown.

**Sequence Name (PSV)**	**Nucleotide position using LC508234-PSapV/46-B/Zambia-2018 as reference (nt)**	**Nucleotide length (nt)**	**Product (Region)**	**IGV Coverage**	**Mapping quality**	**Codon Starts at**	**InDel**
	**Pig45 Colon, BC24**						
MZ515498-PSV-AUS-2018	88-6580	6494	All Polyproteins+ 3D(partial)	3 to 4034	80	362	

MZ515499-PSV-AUS-2018	6686-7498	813	3D Partial	2 to 858	80	3	
	**Pig45 Lung, BC23**						
MZ515500-PSV-AUS-2018	43-6565	6523	All Polyproteins+ 3D(partial)	2 to 948	90	406	
MZ515501-PSV-AUS-2018	6743-7498	756	3D Partial	2 to 124	90	3	
	**Pig46 Colon, BC26**						
MZ515502-PSV-AUS-2018	86-6617	6557	All Polyproteins+ 3D(partial)	3 to 5638	80	364	3 nt insert after 3120 and 21 nt insert after 3123
MZ515503-PSV-AUS-2018	6700-7498	799	3D Partial	2 to 1676	80	1	
	**Pig46 Lung, BC25**						
MZ515504-PSV-AUS-2018	145-2544	2400	L+VP4+2+3+ VP1 (partial)	2 to 76	80	304	
MZ515505-PSV-AUS-2018	2886-3553	668	VP1 (Partial)+ 2A (Partial)	2 to 9	90	2	
MZ515506-PSV-AUS-2018	4191-6112	1923	2C+3A+B+C+ 3D (Partial)	2 to 29	80	2	
MZ515507-PSV-AUS-2018	6593-7496	544	3 D Partial	2 to 20	80	3	

Supplementary Table S3. Twenty two consensus partial sequences of porcine enterovirus-G (PEV-G) were obtained from Pig45 and Pig46 colon and lung swab samples. They were compared with one of the longest obtained sequence MZ515509-EV-G-AUS-2018 of PEV-G and the positions were determined for these partial sequences.

Sequence Name (EV-G)	Nucleotide position using MZ515509-EV-G-AUS-2018 as reference (nt)	Nucleotide length (nt)	Product (Region)	IGV Coverage	Mapping quality Score	Codon starts at	InDel
**Pig45 Colon, BC24**							
MZ515508-EV-G-AUS-2018	197-7189	6993	Complete cds	2 to 3448	80	412-6918	
**Pig45 Lung, BC23**							
MZ515509-EV-G-AUS-2018	1 to 7189	7189	Complete cds	2 to 1686	80	608-7114	
**Pig46 Colon, BC26**							
MZ515510-EV-G-AUS-2018	431-3722	3292	5'UTR-VP4-VP2-VP3-VP1-2A-2B	2 to 243	90	178-3292	
MZ515511-EV-G-AUS-2018	1 to 2746	2746	5'UTR-VP4-VP2-VP3-VP1	2 to 62	80	608-2746	
MZ515512-EV-G-AUS-2018	31-2345	2324	5'UTR-VP4-VP2-VP3	2 to 210	90	578-2324	6 inst after 2095 and 3 inst after 2318
MZ515513-EV-G-AUS-2018	433-2644	2212	5'UTR-VP4-VP2-VP3-VP1	2 to 88	90	176-2212	
MZ515514-EV-G-AUS-2018	4175-6234	2060	2C-3A-3B-3C-3D	2 to 108	90	1	
MZ515515-EV-G-AUS-2018	5432-7189	1758	3C-3D	2 to 162	70	1-1680	

MZ515516-EV-G-AUS-2018	408-1759	1352	5'UTR-VP4-VP2-VP3	2 to 100	90	201-1352	
MZ515517-EV-G-AUS-2018	2749-4026	1278	VP1-2A-2B-2C	2 to 24	80	2	
MZ515518-EV-G-AUS-2018	2729-3924	1199	VP1-2A-2B-2C	2 to 57	90	1	3 inst after 3041
MZ515519-EV-G-AUS-2018	3799-4958	1160	2B-2C-3A	2 to 301	90	2	
MZ515520-EV-G-AUS-2018	4906-6103	1144	2C-3A-3B-3C-3D	2 to 36	90	2	
MZ515521-EV-G-AUS-2018	4158-5271	1114	2C-3A-3B	2 to 193	90	3	
MZ515522-EV-G--AUS-2018	3509-4615	1107	2A-2B-2C	3 to 20	90	1	
MZ515523-EV-G-AUS-2018	5239-6195	957	3C-3D	2 to 77	80	2	
MZ515524-EV-G-AUS-2018	2783-3380	598	VP1-2A	2 to 16	90	1	
**Pig46 Lung, BC25**							
MZ515525-EV-G-AUS-2018	1825-4026	2202	VP3-VP1-2A-2B-2C	2 to 71	90	2	
MZ515526-EV-G-AUS-2018	4029-6058	2030	2C-3A-3B-3C-3D	2 to 123	90	3	
MZ515527-EV-G-AUS-2018	195-1001	807	5'UTR-VP4-VP2	2 to 94	90	414-807	
MZ515528-EV-G-AUS-2018	1061-1813	753	VP2-VP3	2 to 11	90	1	
MZ515529-EV-G-AUS-2018	6458-7055	598	3D Partial	2 to 21	90	1	

Supplementary Table S4. Four and seven partial consensus sequences of porcine teschovirus (PTVs) were obtained from Pig45 and Pig46 colon swab samples, respectively. They were compared with one of the reference sequence AF296088-PTV3/O 2b/GER-2000 of PTV and the positions were determined for these partial sequences.

Sequence Name (PTV)	Nucleotide position using AF296088-PTV3/O 2b/GER-2000 as reference (nt)	Nucleotide length (nt)	Product (Region)	IGV Coverage	Mapping quality	Codon Starts at
	**Pig45 Colon, BC24**					
MZ515530-PTV-AUS-2018	931-1424	494	VP2	2 to 11	80	2
MZ515531-PTV-AUS-2018	4718-5042	325	3A-3B-3C	2 to 5	80	1
MZ515532-PTV-AUS-2018	1957-2271	315	VP3	2 to 20	80	2
MZ515533-PTV-AUS-2018	1426-1689	264	VP2-VP3	3 to 5	80	2
	**Pig46 Colon, BC26**					
MZ515534-PTV-AUS-2018	1962-2669	708	VP3-VP1	2 to 13	80	3
MZ515535-PTV-AUS-2018	2519-3128	610	VP1	2 to 13	80	1
MZ515536-PTV-AUS-2018	3988-4476	489	2C	2 to 10	80	2

MZ515537-PTV-AUS-2018	1959-2383	425	VP3-VP1	2 to 8	64	3
MZ515538-PTV-AUS-2018	3586-3973	388	2B-2C	2 to 8	80	2
MZ515539-PTV-AUS-2018	1549-1882	334	VP2-VP3	2 to 4	80	2
MZ515540-PTV-AUS-2018	1961-2271	311	VP3	2 to 12	80	1

Supplementary Table S5. Ten partial consensus sequences of porcine astrovirus (PAstV) were obtained from Pig46 colon swab sample. They were compared with one of the reference sequence MT470220-PoAstV-CX1-Anhui-China-2018 of PAstV and the positions were determined for these partial sequences.

**Sequence Name (PAstV)**	**Nucleotide position using MT470220-PoAstV-CX1-Anhui-China-2018 as reference (nt)**	**Nucleotide length (nt)**	**Product (Region)**	**IGV Coverage**	**Mapping quality**	**Codon Starts at**	**InDel**
	**Pig46 Colon, BC26**						
MZ515541-PAstV-AUS-2018	5097-6215	1122	ORF2	2 to 16	80	1	3 nt del each after 5321 and 5506, 3 nt insert each after 5969 and 6056, 9 nt insert after 6069 and 6 nt del after 6129
MZ515542-PAstV-AUS-2018	1959-2824	869	ORF1a	2 to 29	80	1 to 691	3 nt insert after 2051
MZ515543-PAstV-AUS-2018	3268-3918	651	ORF1b	3 to 14	80	2	
MZ515544-PAstV-AUS-2018	4902-5392	484	ORF2	2 to 38	80	1	6 nt del after 4968
MZ515545-PAstV-AUS-2018	1605-2049	442	ORF1a	2 to 78	80	1	9 nt del after 1835/ 6 nt insert after 1896
MZ515546-PAstV-AUS-2018	2827-3225	399	ORF1b	2 to 10	80	170	
MZ515547-PAstV-AUS-2018	6428-6716	289	ORF2-3'UTR	2 to 4	80	2 to223	
MZ515548-PAstV-AUS-2018	2268-2548	281	ORF1a	2 to 5	64	1	
MZ515549-PAstV-AUS-2018	4674-4898	225	ORF2	2 to 15	80	1	
MZ515550-PAstV-AUS-2018	305-513	209	ORF1a	2 to 3	80	2	

Supplementary Table S6. Twenty two partial consensus sequences of porcine bocavirus (PBoV) were obtained from Pig45 and Pig46 colon and lung swab samples. They were compared with one of the reference sequence KF025384-PBoV/MN154-1/USA-2011 of PBoV and the positions were determined for these partial sequences.

Sequence Name (PBoV)	Nucleotide position using KF025384-PBoV/MN154-1/USA-2011 as reference (nt)	Nucleotide length (nt)	Product (Region)	IGV Coverage	Mapping quality	Codon Starts at	InDel
	**Pig45 Colon, BC24**						
MZ544028-PBoV-AUS-2018	3511-4294	784	VP1	2 to 750	80	3	
MZ544029-PBoV-AUS-2018	2627-3362	736	NP1-VP1	2 to 33	80	1 to 471 NP1, 461 to 736 VP1	
MZ544030-PBoV-AUS-2018	1748-2203	456	NS1	2 to 48	80	2	
MZ544031-PBoV-AUS-2018	632-1051	420	NS1	2 to 22	80	2	
MZ544032-PBoV-AUS-2018	2749-3097	349	NP1	2 to 46		2-349	
MZ544033-PBoV-AUS-2018	244-542	299	NS1	2 to 6	80	3	
	**Pig45 Lung, BC23**						
MZ544034-PBoV-AUS-2018	2544-4882	2342	NP1-VP1	2 to 888	64	3 to 554 NS1 ,544 to 2342 VP1	6 nt inset after 4341, 6 nt del after 4439, 6 nt insertion after 2043, 3 nt del after 4645,
MZ544035-PBoV-AUS-2018	644-2196	1553	NS1	2 to 83	64	2	
MZ544036-PBoV-AUS-2018	1174-1916	743	NS1	2 to 8	64	3	
MZ544037-PBoV-AUS-2018	3548-4103	559	VP1	2 to 61	64	2	3 insert 3617
MZ544038-PBoV-AUS-2018	2702-3192	491	NP1-VP1	2 to 13	64	1to 396 NP1, 386 to 491 VP1	
	**Pig46 Colon, BC26**						
MZ544039-PBoV-AUS-2018	240-1921	1682	NS1	2 to 180	80	1	
MZ544040-PBoV-AUS-2018	3542-5123	1585	VP1	2 to 268	80	2	3 nt del each after 3607, 4128 and 4635, 6 nt del after 4439, 3 nt insert each after 3763 and 465, 6 nt insert each after 4600 and 4937
MZ544041-PBoV-AUS-2018	3675-4867	1193	VP1	2 to 95	80	1	3 nt insert each after 4132, 4569 and 4598, 6 nt del after 4449 and 3 nt del after 4641
MZ544042-PBoV-AUS-2018	4222-5123	908	VP1	2 to 122	80	3	3 nt insert after 4334, 6 nt insert each after 4569 and 4926, 6 nt del after 4431 and 3 nt del after 4637
MZ544043-PBoV-AUS-2018	1176-1980	805	NS1	2 to 8	80	1	
MZ544044-PBoV-AUS-2018	237-1023	787	NS1	2 to 13	80	1	
MZ544045-PBoV-AUS-2018	3538-4193	650	VP1	2 to 43	80	3	6 nt del after 3588

MZ544046-PBoV-AUS-2018	4417-5061	648	VP1	2 to 14	80	3	6 nt del after 4431, 3 nt del after 4639, 6 nt insert after 4574, 3 nt insert each after 4927 and 4939
MZ544047-PBoV-AUS-2018	1472-1915	444	NS1	2 to 11	80	2	
MZ544048-PBoV-AUS-2018	2411-2738	325	NP1	2 to120	80	1	3 nt insert each after 2446 and 2454, 9 nt del after 2506
	**Pig46 Lung, BC25**						
MZ544049-PBoV-AUS-2018	3540-4119	580	VP1	3 to 34	80	1	

Supplementary Table S7: Three partial consensus sequences of porcine parvovirus 2 (PPV2) were obtained from Pig45 and Pig46 lung swab samples. They were compared with one of the reference sequence MG345013-PPV2/S1/China-2017 of PPV2 and the positions were determined for these partial sequences.

**Sequence Name (PPV2)**	**Nucleotide position using MG345013-PPV2/S1/China-2017 as reference (nt)**	**Nucleotide length (nt)**	**Product (Region)**	**IGV Coverage**	**Mapping quality**	**Codon Starts at**	**Accession number**
	**Pig45 Lung, BC23**						
MZ544050-PPV2-AUS-2018	2316-5301	2986	VP1	2 to 163	64	<1 to 2982	MZ544050
MZ544051-PPV2-AUS-2018	68-1670	1603	5'UTR-NS1	2 to 347	64	110	MZ544051
	**Pig46 Lung, BC25**						
MZ544052-PPV2-AUS-2018	3622-3913	292	VP1	2 to 4	80	<3	MZ544052

Supplementary Table S8. Nine partial consensus sequences of torque teno sus virus k2a (TTSuVk2a) were obtained from Pig45 colon and lung and Pig46 lung swab samples. They were compared with one of the reference sequence MN272073-TTSuVk2a/TTV LV06/Brazil-2016 of TTSuVk2a and the positions were determined for these partial sequences.

**Sequence Name (TTSuVk2a)**	**Nucleotide position using MN272073-TTSuVK2a/TTV_LV06/Brazil-2016 as reference (nt)**	**Nucleotide length (nt)**	**Product (Region)**	**IGV Coverage**	**Mapping quality**	**Codon starts at**	
**Pig45 colon, BC24**							
MZ544053-TTSuVk2a--AUS-2018	1945-2445	501	ORF1	2 to 11	90	1 to 401	
MZ544054-TTSuVk2a-AUS-2018	1416-1774	359	ORF1	2 to 11	90	2	
MZ544055-TTSuVk2a-AUS-2018	339-646	308	ORF2 and ORF1	2 to 7	90	66 to 275 ORF2, 149 to 308 ORF1	
MZ544056-TTSuVk2a-AUS-2018	706-942	237	ORF1	2 to 4	90	1	
PC45-BC-24-TTSuVk2a-1184-1334-AUS-2018	1184-1334	151		2 to 7	90		
**Pig45 Lung, BC23**							

MZ544057-TTSuVk2a-AUS-2018	1174-2449	1276	ORF1	3 to 74	90	1 to 1176	
MZ544058-TTSuVk2a-AUS-2018	206-1071	866	ORF2 and ORF1	2 to 44	90	199 to 408 ORF2, 282 to 866 ORF1	
**Pig46 Lung, BC25**							
PL46-BC-25-TTSuVk2a-1416-1562-AUS-2018	1416-1562	147	ORF1	2	90		
PL46-BC-25-TTSuVk2a-2224-2340-AUS-2018	2224-2340	117	ORF1	2	90		

Supplementary Table S9. One complete coding sequence (cds) torque teno sus virus k2b TTSuVk2b was obtained from Pig45 lung swab and four partial consensus sequences of TTSuVk2b were obtained from Pig45 colon and Pig46 lung swab samples. The longest obtained sequence MZ544061-TTSuVk2b-AUS-2018 was used to determine the given positions for the other short partial sequences.

**Sequence Name (TTSuVk2b)**	**Nucleotide position using MZ544061-TTSuVk2b-AUS-2018as reference (nt)**	**Nucleotide length (nt)**	**Product (Region)**	**IGV Coverage**	**Mapping quality**	**Codon Starts at**	
**Pig45 Colon, BC24**							
MZ544059-TTSuVk2b-AUS-2018	1335-2511	1177	ORF1 and ORF3	2 to 51	64	607-1042 ORF3, 1-1065 ORF1	
MZ544060-TTSuVk2b-AUS-2018	279-1214	936	ORF1, ORF2 and ORF3	2 to 86	64	101-343 ORF2, 232-936 ORF1, 101-339 ORF3	
**Pig45 Lung, BC23**							
MZ544061-TTSuVk2b-AUS-2018	1 to 2565	2565	Complete cds	2 to 355	64	379-621 ORF2, 510-2399 ORF1, 379-617, 1941-2376 ORF3	
**Pig46 Lung, BC25**							
MZ544062-TTSuVk2b-AUS-2018	661-1057	397	ORF1	2 to 9	64	3 to 397	
MZ544063-TTSuVk2b-AUS-2018	1659-1861	203	ORF1	2 to 6	64	1 to 203	

Supplementary Figure S1

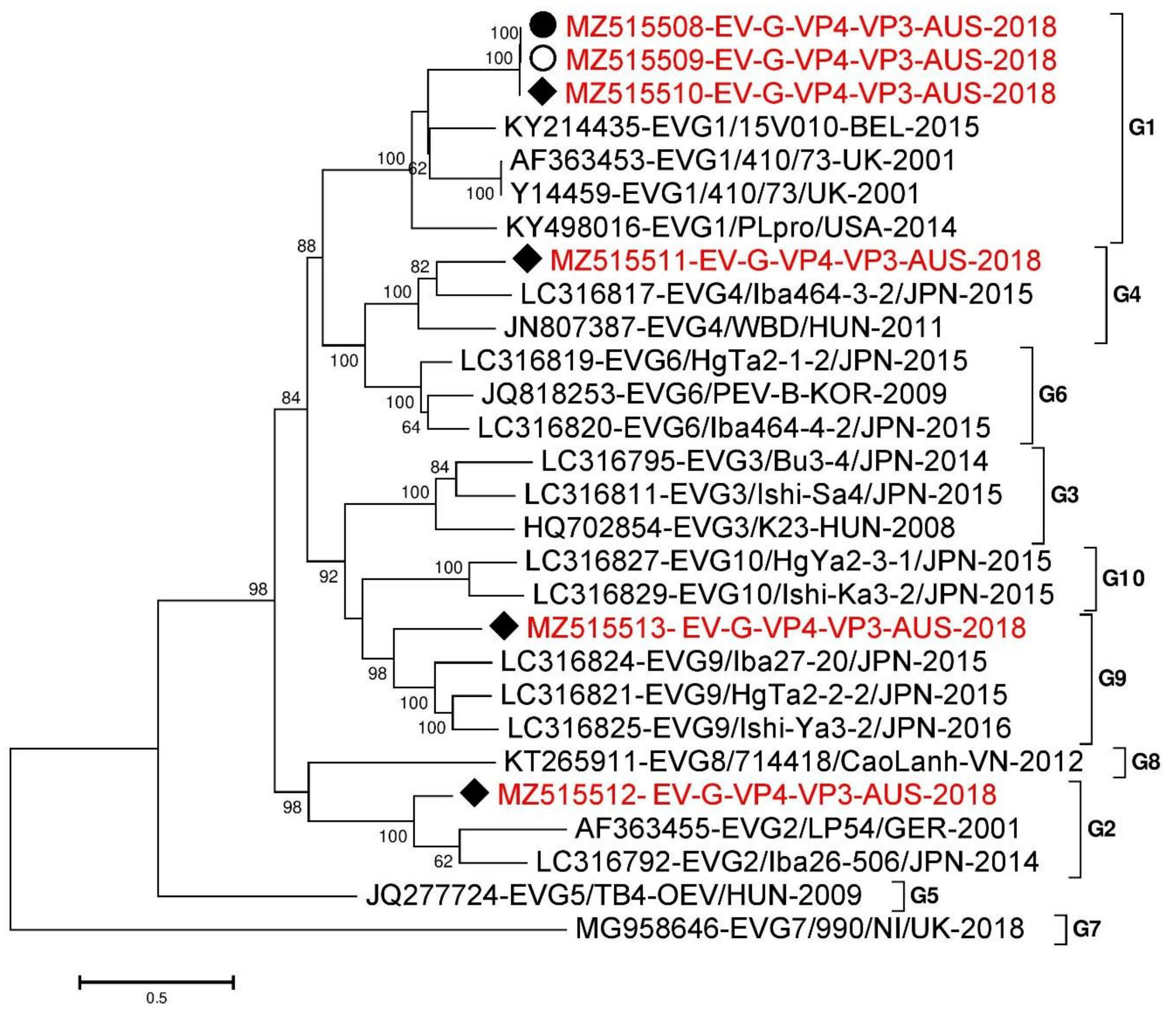

Supplementary Figure S2

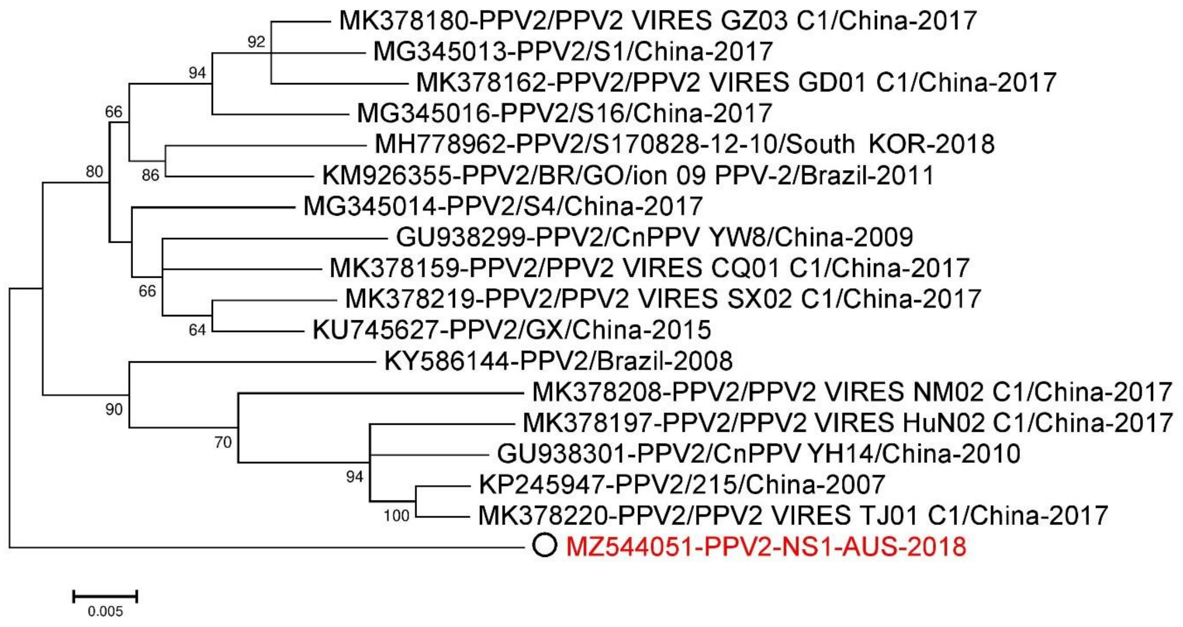

Supplementary Figure S3

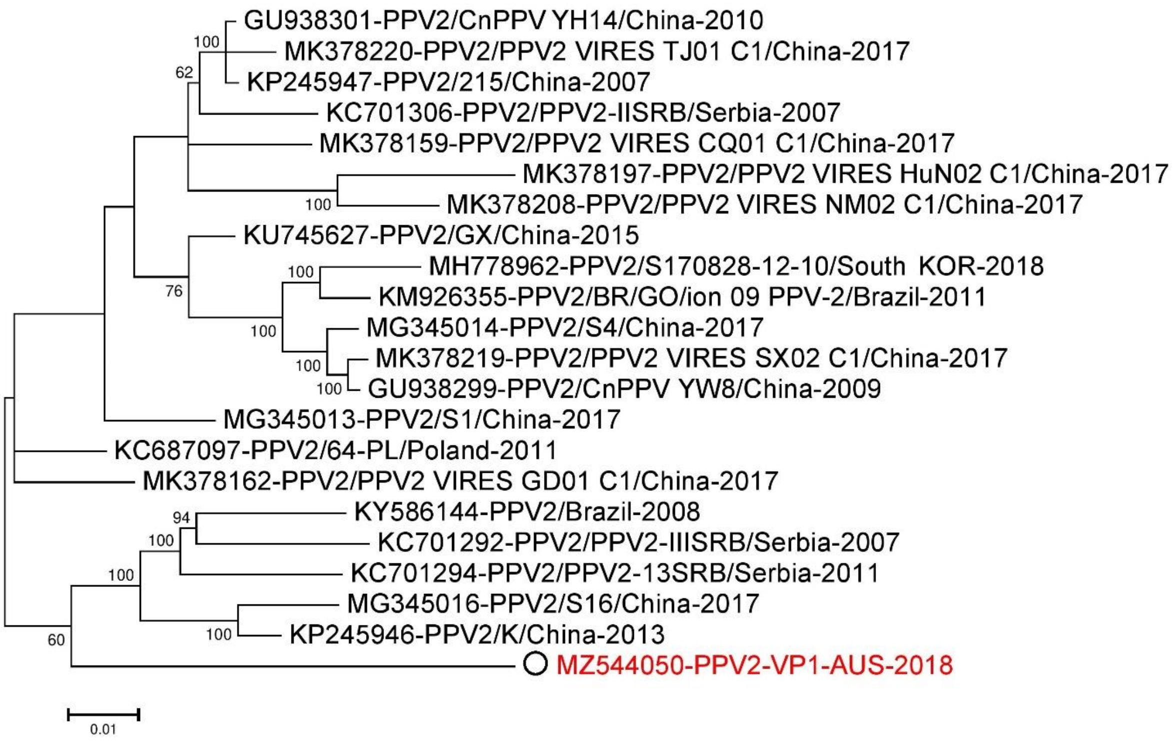

Supplementary Figure S4

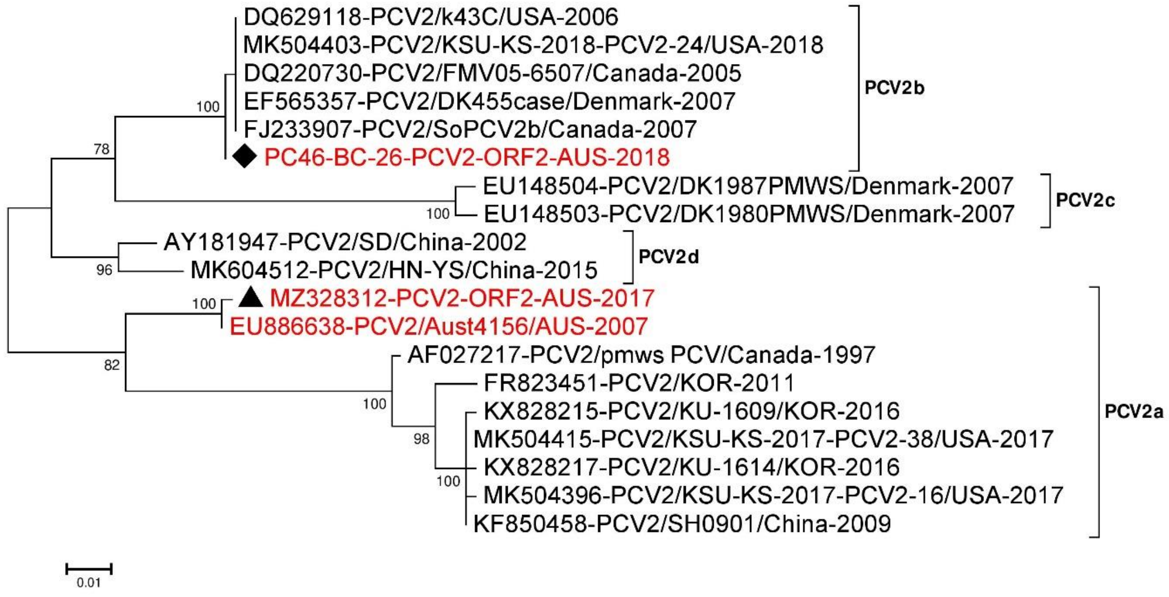

Supplementary Figure S5

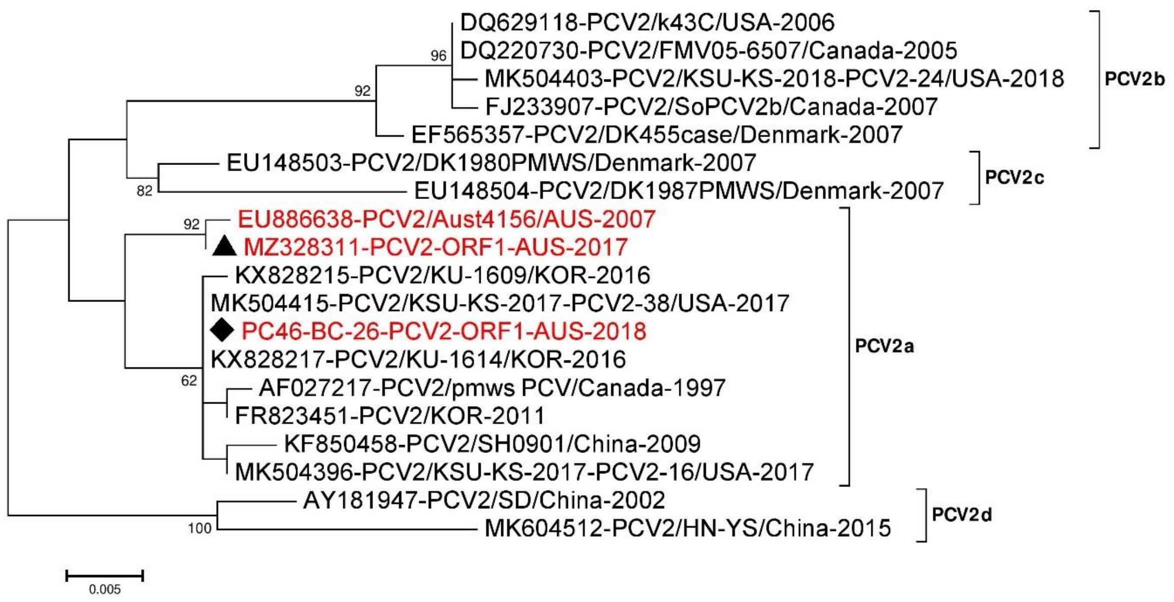
DQ629118-PCV2/k43C/USA-2006
DQ220730-PCV2/FMV05-6507/Canada-2005
MK504403-PCV2/KSU-KS-2018-PCV2-24/USA-2018
FJ233907-PCV2/SoPCV2b/Canada-2007
EF565357-PCV2/DK455case/Denmark-2007
EU148503-PCV2/DK1980PMWS/Denmark-2007
EU148504-PCV2/DK1987PMWS/Denmark-2007
EU886638-PCV2/Aust4156/AUS-2007
MZ328311-PCV2-ORF1-AUS-2017
KX828215-PCV2/KU-1609/KOR-2016
MK504415-PCV2/KSU-KS-2017-PCV2-38/USA-2017
PC46-BC-26-PCV2-ORF1-AUS-2018
KX828217-PCV2/KU-1614/KOR-2016
AF027217-PCV2/pmws PCV/Canada-1997
FR823451-PCV2/KOR-2011
KF850458-PCV2/SH0901/China-2009
MK504396-PCV2/KSU-KS-2017-PCV2-16/USA-2017
AY181947-PCV2/SD/China-2002
MK604512-PCV2/HN-YS/China-2015
PCV2b
PCV2c
PCV2a
PCV2d
96
92
82
92
62
100
0.005
