# Supplementary material for: Exploring the Cause of Diarrhoea and Poor Growth in 8–11-Week-Old Pigs from an Australian Pig Herd Using Metagenomic Sequencing
Source: Viruses. 2021 Aug 13;13(8):1608. doi: 10.3390/v13081608 (PMC8402840; doi:10.3390/v13081608)
Supplement: Supplementary file 1 [file viruses-13-01608-s001.zip › Supplementary Data 1 Porcine Viruses Manuscript 22 July 2021.pdf]

Supplementary Data 1

Exploring the cause of diarrhoea and poor growth in 8-11 weeks old pigs from an Australian pig herd using metagenomics sequencing

Tarka Raj Bhatta [1,2], Anthony Chamings [1,2] , Soren Alexandersen*[1,2,3]

[1]Geelong Centre for Emerging Infectious Diseases, Geelong, VIC 3220, Australia; [2]Deakin University, School of Medicine, Geelong, VIC 3220, Australia; [3]Barwon Health, Geelong, VIC 3220 Australia

*Corresponding Author: soren.alexandersen@deakin.edu.au

```
>PCV2 Conjugated 40 PC46 BC26
ACGTCATATCTGAAAACGAAAGAAGTGCGCTGTAAGTATTACCAGCGCACTTCGGCAGCGGC
AGCACCTCGGCAGCACCTCAGCAGCAACATGCCCAGCAAGAAGAATGGAAGAAGCGGACCCC
AACCACATAAAAGGGGGGTGTTCACTCTGAATAATCCTTCCGAAGACGAGCGCAAGAAAATA
CGGGAGCTCCCAATCTCCCTGTTTGATTATTTTATTGTTGGCGAGGAGGGTAATGAGGAAGG
ACGAACACCTCACCTCCAGGGGTTCGCTAATTTTGTGAAGAAGCAAACTTTTAATAAAGTGA
AGTGGTATTTGGGTGCCCGCTGCCACATCGAGAAAGCCAAAGGAACTGATCAGCAGAATAAA
GAATATTGCAGTAAAGAAGGCAACTTACTTATTGAATGTGGAGCTCCTCGATCTCAAGGACA
ACGGAGTGACCTGTCTACTGCTGTGAGTACCTTGTTGGAGAGCGGGAGTCTGGTGACCGTTG
CAGAGCAGCACCCTGTAACGTTTGTCAGAAATTTCCGCGGGCTGGCTGAACTTTTGAAAGTG
AGCGGGAAAATGCAGAAGCGTGATTGGAAGACCAATGTACACGTCATTGTGGGGCCACCTGG
GTGTGGTAAAAGCAAATGGGCTGCTAATTTTGCAGACCCGGAAACCACATACTGGAAACCAC
CTAGAAACAAGTGGTGGGATGGTTACCATGGTGAAGAAGTGGTTGTTATTGATGACTTTTAT
GGCTGGCTGCCGTGGGATGATCTACTGAGACTGTGTGATCGATATCCATTGACTGTAGAGAC
TAAAGGTGGAACTGTACCTTTTTTGGCCCGCAGTATTCTGATTACCAGCAATCAGACCCCGT
TGGAATGGTACTCCTCAACTGCTGTCCCAGCTGTAGAAGCTCTCTATCGGAGGATTACTTCC
TTGGTATTTTGGAAGAATGCTACAGAACAATCCACGGAGGAAGGGGGCCAGTTCGTCACCCT
TTCCCCCCCCTGCCCTGAATTTCCATATGAAATAAATTACTGAGTCTTTTTTTATCACTTCG
TAATGGTTTTTATTATTCATTAAGGGTTAAGTGGGGGGTCTTTAAGATTAAATTCTCTGAAT
TGTACATACATGGTTACACGGATATTGTATTCCTGGTCGTATATACTGTTTTCGAACGCAGT
GCCGAGGCCTACGTGGTCTACATTTCCAGCAGTTTGTAGTCTCAGCCACAGCTGGTTTCTTT
TGTTGTTTGGTTGGAAGTAATCAATAGTGGAATCTAGGACAGGTTTGGGGGTGAAGTAGCGG
GAGTGGTAGGAGAAGGGCTGGGTTATGGTATGGCGGGAGGAGTAGTTTACATAGGGGTCATA
GGTGAGGGCTGTGGCCTTTGTTACAAAGTTATCATCTAGAATAACAGCACTGGAGCCCACTC
CCCTGTCACCCTGGGTGATCGGGGAGCAGGGCCAGAATTCAACCTTAACCTTTCTTATTCTG
TAGTATTCAAAGGGCACAGAGCGGGGGTTTGAGCCCCCTCCTGGGGGAAGAAAGTCATTAAT
ATTGAATCTCATCATGTCCACCGCCCAGGAGGGCGTTCTGACTGTGGTTCGCTTGATAGTAT
ATCTGAAGGGGCGGGGGAGGCGGGTATTGAAGATGCCGTTTTTCCTTCTCCAGCGGTAACGG
TGGCGGGGGTGGACGAGCCAGGGGCGGCGGCGGAGGATCTGGCCAAGATGGCTGCGGGGGCG
GTGTCTTCTTCTCCGGTAACGCCTCCTTGGATACGTCATATCTGAAAACGAAAGAAGTGCGC
TGTAAGTATTACCAGCGCACTTCGGCAGCGGCAGCACCTCGGCAGCACCT
```
